# Two-Component Systems in *Pasteurellaceae* and Their Roles in Virulence

**DOI:** 10.3390/vetsci12121140

**Published:** 2025-11-29

**Authors:** Eduardo M. Martin, Alma L. Guerrero-Barrera, F. Javier Avelar-Gonzalez

**Affiliations:** 1 Laboratorio de Biología Celular y Tisular, Departamento de Morfología, Universidad Autónoma de Aguascalientes, Aguascalientes 20131, Mexico; mauricio.martin@edu.uaa.mx; 2 Laboratorio de Estudios Ambientales, Departamento de Fisiología y Farmacología, Universidad Autónoma de Aguascalientes, Aguascalientes 20131, Mexico; javier.avelar@edu.uaa.mx

**Keywords:** *Pasteurellaceae*, two-component systems, signal transduction, transcriptional regulation, virulence regulation, comparative genomics, regulatory networks, computational modeling

## Abstract

**Simple Summary:**

Bacteria survive by detecting changes around them and adjusting their behavior accordingly. To do this, they use protein pairs that act like on/off switches, known as two-component systems. When activated by a specific signal, these systems trigger a response that helps bacteria tolerate stress or cause infection. This review examines how these systems function in members of the *Pasteurellaceae* family and how even closely related species can respond very differently to similar signals. The review summarizes those systems that are best understood, where knowledge gaps remain, and how differences in gene regulation may explain variations in virulence. New computational approaches—including artificial intelligence, molecular modeling, molecular dynamics, and network analysis—that can predict how these protein switches interact and control bacterial behavior are also highlighted. Understanding these signaling systems may help identify new drug targets and improve strategies to prevent or treat infections caused by these and other pathogens.

**Abstract:**

Two-component systems (TCSs) are widespread in bacteria and archaea, with only limited presence in eukaryotes. These signaling mechanisms detect environmental changes and adjust gene expression to survive and adapt. In this review, TCSs were examined within the *Pasteurellaceae* family, focusing on how closely related organisms employ similar systems to regulate infections and stress responses. Comparative analysis revealed that homologous TCSs can differ markedly in the signals they detect and in the genes or virulence factors they control, underscoring the functional diversity that has evolved even within this family. Inconsistencies in nomenclature across studies are also identified, which complicate data integration and cross-species comparisons. Given these challenges, the need for unified naming conventions and broader, system-level analyses is highlighted. It is further proposed that emerging computational tools—including molecular modeling, molecular dynamics, and neural network-based analyses—offer powerful strategies to predict signaling interactions, identify conserved regulatory models, and clarify how these systems contribute to bacterial adaptation and pathogenicity.

## 1. Introduction

**Figure 1:**
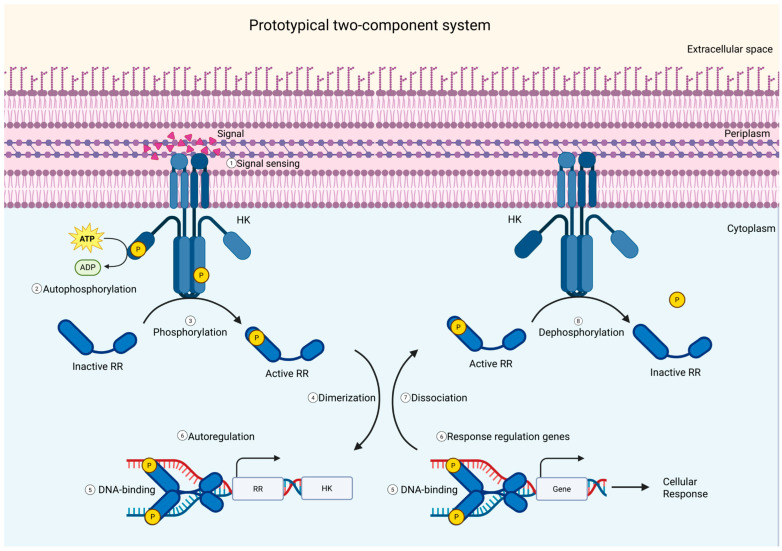
Prototypical two-component system. (1) A membrane-bound histidine kinase (HK) detects environmental signals through its sensor domain and (2) autophosphorylates a conserved histidine residue. (3) The phosphoryl group is then transferred to an aspartate residue on the cognate cytoplasmic response regulator (RR). (4, 5) The phosphorylated RR undergoes a conformational change that activates or represses transcription of target genes, (6) allowing the cell to adapt to environmental changes. (7, 8) Once the signal subsides, RR dimers dissociate, and the HK functions as a phosphatase to dephosphorylate the RR, thereby terminating the response and resetting the system for subsequent signaling events. Many HKs and RRs form specific cognate pairs, but cross-regulation and signal integration may occur between non-cognate partners in more complex regulatory networks.

Two-component systems (TCSs) sense and respond to changes in the bacterial environment through signal transduction mechanisms; in their prototypical form, these are composed of a sensor and a response protein. Histidine kinases (HKs) autophosphorylate upon recognition of a specific stimulus and transfer a phosphoryl group to their cognate response regulators (RRs), switching them, in most cases, to an active transcriptional state [1,2]. The specific recognition between cognate histidine kinases and response regulators is then a determinant for the coupling of external stimuli to particular and adequate cellular responses. As soon as a stimulus has been recognized, the autophosphorylated HK must discriminate its cognate RR from other non-cognate substrates before transferring the phosphoryl group (Figure 1) [3,4]. There are three primary mechanisms for ensuring TCS pathway specificity: molecular recognition, phosphatase activity, and substrate competition; additionally, spatial–temporal regulation could also be involved, but it has not been thoroughly assessed [5,6,7,8,9,10]. The predominant mechanism enforcing specificity is molecular recognition, the intrinsic ability of an autophosphorylated HK to recognize its cognate partner through interfacial surface interactions [8,11,12].

The family *Pasteurellaceae* consists of 65 named species [13] in 25 genera, and it is a highly heterogeneous group of Gram-negative bacteria that has undergone numerous reclassifications [14]. By comparison, the *Pasteurellaceae* website lists 35 genera with 109 organisms [15]. Many members present a delicate balance between commensal and pathogenic lifestyles in vertebrate hosts. Species belonging to this family often reside as non-pathogenic flora in the upper respiratory tract and oral cavity, but under certain conditions, this relationship can shift to a pathogenic one, resulting in disease. The mechanisms underlying this switch are complex and not well understood, involving both host and bacterial factors [13].

Some species have broad host ranges, including humans, while others are restricted to a single host species (i.e., pigs, ruminants, birds, or humans). Several members of the family are clinically and economically important pathogens of humans and animals [14].

Representative members, their hosts, and associated infections are summarized in Table 1. This article focuses on some of the most important pathogens affecting either animal or human health, highlighting virulence factors in these organisms that are associated with two-component systems.

**Table 1:** Overview of selected *Pasteurellaceae* species, host ranges, and associated diseases. Species included represent common opportunistic pathogens infecting humans or livestock. The table summarizes major hosts, clinical presentations, and infection types referenced throughout the comparative analysis of two-component systems.

Organism	Host(s)	Disease(s)	Infection Type	References
*Haemophilus (H.) influenzae*	Human	Bacteremia, respiratory tract infections, meningitis, sinusitis, otitis media, conjunctivitis	Opportunistic	[16,17,18,19,20,21]
*Actinobacillus (A.) pleuropneumoniae*	Swine	Porcine pleuropneumonia	Opportunistic	[22,23,24,25,26,27,28]
*Glaesserella (G.) parasuis*	Swine (Piglets)	Pneumonia, Glässer disease	Opportunistic	[29,30,31,32,33,34]
*Mannheimia (M.) haemolytica*	Cattle, sheep, other ruminants.	Fibrinous, necrotic pneumonia	Opportunistic	[35,36,37,38]
*Pasteurella (P.) multocida*	Avian, ungulates, swine, rabbits, humans, wild animals	Fowl cholera, pneumonia, atrophic rhinitis, hemorrhagic septicemia	Opportunistic	[39,40,41,42]

The selected species are well-studied representatives of the *Pasteurellaceae* family, with sequenced genomes and documented TCSs. They infect a broad range of hosts, enabling comparison of conserved and divergent regulatory responses across different species. These pathogens cause substantial economic losses in animal production and significant morbidity in human populations, making their virulence regulation and environmental sensing highly relevant for both veterinary and medical microbiology.

## 2. Known TCSs in Key Pathogens

**Figure 2:**
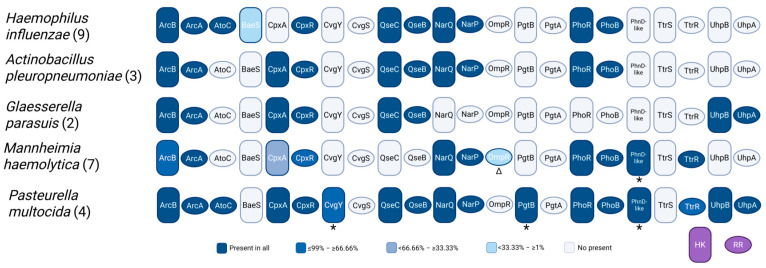
Distribution and conservation of two-component systems (TCSs) across representative *Pasteurellaceae* species. Rounded rectangles represent histidine kinases (HKs), and ovals represent response regulators (RRs). Color intensity indicates conservation across available genomes of each species. Numbers in parentheses denote the number of genomes analyzed per species according to the prokaryote two-component system (P2CS) repository [44]. Asterisks indicate proteins with conflicting annotations, and Δ denotes partial protein sequences.

The distribution outlined below also underscores the need to revisit TCS classification (Figure 2). Current repositories (e.g., NCBI and P2CS) often differ in their annotation pipelines and classification methods [43,44]. With the increasing availability of multispecies records in NCBI, earlier classification may no longer capture the full diversity of these system proteins. In addition, the AlphaFold protein structure database now provides modeled structures for many TCS proteins [45], which could be leveraged as an independent criterion for family assignment to complement sequence-based methods. A central question moving forward is whether TCSs should be named primarily according to their experimentally defined functions or based on homology at the sequence or structural level—or a combination of both. Establishing a consistent framework will be essential for comparing TCSs across taxa to better understand their regulatory networks and function.

The number of classified or annotated proteins may differ from NCBI entries due to differences in annotation pipelines [43]. The number of histidine kinases (HKs) and response regulators (RRs) varies among species, with *P. multocida* showing the largest repertoire. Core systems such as ArcBA, NarQP, and PhoRB are broadly conserved and are typically involved in sensing fundamental environmental cues such as oxygen availability, nitrate levels, and phosphate restriction [46]. These systems control essential metabolic and stress-response pathways and therefore tend to be retained across *Pasteurellaceae*. In contrast, others (e.g., CpxAR, CvgSY, BaeS) occur sporadically. ArcB, NarQ, QseC, and PhoR appear to be duplicated in *H. influenzae* KR494, but these duplicates are annotated under the *H. influenzae* R2866 genome in NCBI.

## 3. Mechanisms of Virulence Regulation

TCSs are key regulators of virulence across *Pasteurellaceae*, allowing bacteria to sense host-associated cues and adjust gene expression for survival, colonization, and immune evasion. Their regulatory targets commonly include genes involved in capsule biosynthesis, lipooligosaccharide modification, pilus production, iron intake, stress tolerance, and biofilm formation. Although some systems are conserved, their regulatory targets and cascades can differ between species, reflecting niche adaptation and host specificity. The following sections summarize the main TCSs characterized and highlight how the regulatory outputs contribute to infection and disease, based on available transcriptomic, phenotypic, and genetic studies.

### 3.1. Haemophilus influenzae

#### 3.1.1. ArcBA

The virulence of the *arcA* single mutant was compared to that of the *tonB* knockout; both showed reduced virulence and lower medium lethal doses compared to the wildtype parent strain (*H. influenzae* type b ATCC 10211), hereinafter called Hib 10211. No differences were detected in lipopolysaccharide synthesis, type b capsule production, or adherence to Hep-2 cells between the *arcA* mutant and Hib 10211. Adherence assays were performed under both aerobiosis and anaerobiosis [16].

In non-typeable *H. influenza* (NTHI) clinical isolate (NT127), the lipooligosaccharide (LOS) glycosyltransferase-related genes *lic2B* and *lic2C* are regulated by ArcA under anaerobic conditions. Lic2C adds a proximal glucose in an *a*1-3 linkage to heptose II (Hep II) in the LOS inner core. Lic2B adds a *b*-galactose at position 4 of an *a*-glucose extension to Hep II in the LOS outer core, a role elucidated by the authors. Both proteins are important for serum resistance in NTHI. This redox-state regulation of LOS-related genes links classic virulence factors to physiological responses that support persistent colonization of the human respiratory tract or pathogenesis by NTHI [17].

Two *arcA* mutants displayed hypersensitivity to human serum compared to parental Hib 10211 or complemented strains. Because hemin is required for aerobic growth of the organism, serum was supplemented with hemin, but this did not rescue survival. The observed hypersensitivity may result from the absence of proteins required for utilization of electron donors other than molecular oxygen [16]. Likewise, an *arcA* mutant exposed to 3% serum killed around 60% of the bacteria in contrast to the parental NT127. Under aerobic conditions, no difference was observed. Mutant sensitivity was attributed to iC3b, a cleavage product of complement component factor C3b [17].

Two-dimensional gels under aerobic and anaerobic conditions were run to identify genes regulated by ArcA. Aerobic expression was so indistinguishable between the *arcA* mutant and the parental strain that no further analysis was conducted, whereas global gene expression under anaerobic conditions was evidently different. The *arcA* mutant profile had many protein spots that were either totally absent or had much lower intensities when compared to the parental strain protein profile; consequently, the parental profile contained several spots that were either absent or had lower intensities in the mutant profile. The full ArcA regulon was not analyzed in detail, but some regulated membrane proteins could be identified. It was found that ArcA seemed to be responsible for the oxygen-dependent regulation of formate dehydrogenase and fumarate reductase. Interestingly, L-lactate dehydrogenase is expressed under both aerobic and anaerobic conditions [16].

The ArcA regulon was revisited and characterized further by Wong et al. [18] using microarray analysis comparing the *arcA* single mutant to the wildtype parental strain (*H. influenza* Rd). Seven genes showed increased expression or upregulation and were related to the respiratory chain (*fdxH*, *fdxI*, *fdhE*, *ndh*, and *lldD*) and TCA cycle (*sucA* and *sucB*). Some of these have already been described in DeSouza et al. [16], such as *fdxH* and *fdxI*, which are the *b* and *g* subunits of formate dehydrogenase, respectively, and *fhdE*, an accessory protein needed to form formate dehydrogenase, as well as *lldD*, which is L-lactate dehydrogenase. In the same analysis, downregulated genes were *arcA*, a hypothetical protein; ornithine decarboxylase (*speF*), a *dps*-like protein; and putrescine–ornithine antiporter (*potE*), the response regulator ArcA positive feedback is associated with autoregulation in an input-stimulus-dependent fashion [47,48]. Dps proteins are related to oxidative stress, iron binding, and Fe(II) oxidation with H_2_O_2_ to form the stable ferric oxide mineral, avoiding the formation of toxic hydroxyl radicals. Consistent with the latter, a *dps* single mutant showed increased sensitivity to H_2_O_2_ following anaerobic growth and lower sensitivity in aerobic conditions [18].

The authors suggest that as this organism transits from low- to high-oxygen environments within the host, the ArcBA system protects the bacteria against oxidative stress via a molecular mechanism for resistance to reactive oxygen intermediates (ROIs) generated by this system [18].

#### 3.1.2. QseCB

In the clinical respiratory isolate strain NTHI 2019, QseCB homologs respond to cold temperatures, ferrous iron, and zinc, but no ferric iron or other cations. Based on these results, the authors proposed renaming the system to ferrous iron-responsive regulator/sensor (FirRS). However, given that this proposal was based on a single strain and the system falls within the broader QseCB family, the current naming convention will be used (Table 3).

In parental NTHI 2019, exposure to 9 °C increased expression of the operon *ygiW-qseBC*, whereas single *qseC* and *qseB* mutants failed to activate its expression, indicating autoregulation of the operon. Expression assays further showed that *qseBC* was not induced by epinephrine (EPI), norepinephrine (NE), or serotonin in concentrations up to 200 mM, and no response to AI-3 was detected [19]. Moreover, deletion of *qseC* in clinical isolate NTHI Hi3655 did not alter autoinducer-2 (AI-2) production compared to its parental strain [20]. Taking the results together, a specific signaling molecule for this system is yet to be identified, if one exists. Nevertheless, AI-2 has been reported as a quorum signal in the related strain NTHI 86-028NP [49].

A *qseC* mutant showed reduced biofilm formation in both static and flow conditions compared with parental Hi3655. This suggests that QseC contributes to biofilm regulation, possibly through control of adhesin or LOS-biosynthesis genes. Although further work is needed to clarify the role of QseC during biofilm formation, it has been proposed that QseC may mediate a switch from a biofilm to a non-biofilm phenotype in response to an as-yet unidentified signal [20].

In NTHI 2019, *qseBC* expression was induced by FeCl_2_ and, to a lesser extent, ZnCl_2_, whereas FeCl_3_ and reactive radicals did not activate the system. A YwiG-GFP reporter showed reduced expression in both *qseC* and *qseB* mutants in response to FeCl_2_. Complementation restored expression, though *qseB* complementation resulted in underexpression while *qseC* complementation caused overexpression, suggesting that the ferrous iron-dependent activation of *ygiW-qseBC* occurs primarily through QseC. The dual function of QseC was shown to be independent, as double point mutations Y149G and R150T abolished the response to Fe^2+^ while leaving the cold-shock response intact. QseB was also required, as the point mutation D51A prevented *ygiW* transcription in response to FeCl_2_ [19]. Finally, virulence assays demonstrated that infection was attenuated in mice challenged with ygiW, *qseB*, or *qseC* single mutants compared with the wildtype strains NTHI 2019 or NTHI 86-028 [19].

#### 3.1.3. NarQP

In *H. influenzae* Rd strain KW20, the *narP* mutant showed growth deficiencies when grown with DMSO or fumarate as an electron acceptor compared to the parental KWD20. When nitrate was used as the electron acceptor, the *narP* mutant grew very slowly, while the complemented strain grew relatively well. It was also demonstrated that NarP was required for nitrate induction of periplasmic nitrate reductase synthesis [21].

A conserved target of the NarQP system, present in both *E. coli* and *H. influenzae*, is the *napFDAGHBC* operon, which encodes the cytochrome *c*-linked nitrate reductase. Using the upstream sequence of *napF* in an *E. coli* background, it was shown that this promoter could be regulated by anaerobiosis and by NarP and NarL. NarP and NarL regulate this region in response to nitrate and nitrite, respectively. Phosphorylated NarP acts as an activator, while phosphorylated NarL antagonizes expression from this region.

Additionally, NarP shows a synergetic relation with the fumarate and nitrate reductase protein (Fnr), which controls several operons during anaerobic growth—an architecture that may be broadly representative of the *g*-proteobacteria [21]. Unfortunately, the specific regulatory targets of the NarQP system in *H. influenzae* remain unknown, and further research is needed to identify them.

### 3.2. Actinobacillus pleuropneumoniae

#### 3.2.1. ArcBA

In *A. pleuropneumoniae* 7 AP76, a pathogen exclusive to pigs, an *arcA* partial deletion mutant showed attenuated infection persistence, deficient autoaggregation under anaerobic conditions, and altered biofilm formation as early as 18 h after inoculation under anaerobic growth conditions. However, no significant differences were observed in survival or growth under either aerobic or anaerobic conditions. Microarray analysis comparing an *arcA* deletion with a wildtype strain under anaerobic conditions revealed that *arcA* positively regulated its own transcription, as well as *narQ* and *cpxR*, thereby possibly inducing the *cpxRA* operon. Conversely, *arcA* downregulates *aceE*, *aceF*, and *lpdA*, encoding subunits from the pyruvate dehydrogenase complex, which were downregulated ~3-fold at the protein level. This suggests pyruvate accumulation due to reduced conversion to Acetyl-CoA. Under anaerobic conditions, *arcA* downregulates the *aldA* and *adhI* transcripts by ~17- and ~5-fold, respectively, and their protein products AldA and AdhI by ~4- and ~3-fold, both involved in the two-step fermentation of acetyl-CoA to ethanol. In contrast, p*flB* and *ackA* were upregulated by ~1.4- and ~1-fold, encoding enzymes catalyzing conversions of pyruvate to acetyl-CoA and acetyl-phosphate to acetate, respectively [22,23].

#### 3.2.2. CpxAR

Transcriptomic analysis of the *cpxAR* mutant revealed 265 differentially expressed genes, with 134 upregulated and 131 downregulated compared to wildtype *A. pleuropneumiae* serovar 1 S4074 at 42 °C. Among the upregulated genes were several type IV pilus-associated genes (*apfA*, *apfB*, *apfC*, *apfD*, *pilM*, *pilN*, *pilO*, *pilP*, and *pilQ*), which were induced at both 37 °C and 42 °C. This suggests that CpxAR represses pilus gene expression independently of temperature, and that this system-mediated heat stress is connected to overexpression of pilus proteins. Although these nine pilus genes are crucial for fever-like temperature resistance, only the *cpxAR* and *apfA* mutants maintained normal growth at 42 °C [24].

The system also regulates capsule biosynthesis. In a *cpxAR* mutant, expression of four capsular polysaccharide export (CPS) genes (*cpxD*, *cpxC*, *cpxB*, and *cpxA*) was decreased, showing that they are directly regulated by CpxR. CPS is one of the major virulence factors that protects bacteria from phagocytic and complement-mediated bacteriolysis by the host. Both *cpxAR* and *cpxD* mutants lacked layers of capsular material, and infection experiments demonstrated attenuated virulence. Mice infected with these mutants had survival rates of 83% and 100%, respectively, compared to the low survival in wildtype or complemented strains. Consistently, bacterial loads in lungs and liver were reduced, and pathologies such as pneumonia, hyperemia, edema, and consolidation were absent in infected mice. Together, these results demonstrate that the CpxAR *cpxDCBA* pathway contributes to capsule biosynthesis and pathogenesis [25].

CpxAR also contributes to adhesion and metabolism. During adherence of *A. pleuropneumoniae* to Saint-Jude Porcine Lung cells, *cpxR* and *ackA* transcripts were downregulated ~2- and ~3-fold, respectively, inhibiting central metabolic response and CpxAR protein synthesis [26]. In contrast, during acute infection, *cpxA* is upregulated [27]. In field isolates forming biofilm under sub-inhibitory concentrations of Penicillin G, *cpxRA* was also differentially regulated during biofilm formation: *cpxA* was upregulated in a 4 h static biofilm, whereas *cpxR* was downregulated in drip-flow conditions [28,50].

Finally, CpxAR contributes to stress resistance beyond capsule biosynthesis. A *cpxAR* deletion demonstrated that CpxAR directly regulates *wecA*, which is co-transcribed, revealing that the system is involved in O-antigen biosynthesis. Both *wecA* and *cpxAR* mutants showed reduced porcine serum resistance, decreased tolerance to H_2_O_2_ and NaCl, and attenuated virulence in a mouse infection model, including reduced colonization and milder pathology compared to the wild type [51].

#### 3.2.3. NarQP

*Actinobacillus pleuropneumoniae* possesses an incomplete denitrification pathway and a complete dissimilatory reduction pathway, encoded by nitrate reductase (*napFDAGHBC*), nitrite reductase (cytochrome C_552_) Nrf (*nrfABCD*), and nitrite reductase NirK (*nirK*). The optimal supplementation of NaNO_3_ for growth promotion was 5 mM, and it accelerated growth under anaerobic conditions compared to aerobic ones [52]. The gene *narQ* is upregulated during anaerobic stress [53], consistent with its role in coordinating nitrate-dependent growth advantages under hypoxic conditions. Although mortality of infected mice was not significantly different between the wild type (4070) and the nitrate-supplemented group, mortality did increase with higher nitrate concentration. Thus, nitrate can enhance bacterial growth in vitro and lethality in vivo [52].

Transcriptomic analysis under anaerobic conditions with nitrate supplementation revealed 112 upregulated and 189 downregulated genes. Pathway enrichment analysis showed that metabolic pathways were the most affected, representing 75.9% of the upregulated genes and 56.8% of the downregulated genes, while environmental processing pathways represented 14.5% of the upregulated genes and 13.6% of the downregulated genes. Activation of metabolic pathways in a nitrate-rich context suggests that *A. pleuropneumoniae* alters its metabolic network to obtain energy from multiple substrates. These findings indicate that, in a hypoxic environment, nitrate enhances energy production by stimulating nitrate metabolic pathways involved in carbohydrate, lipid, and energy metabolism, thereby promoting growth. The related nitrate growth advantage was absent under anaerobiosis in the *narQ* and *narP* mutants. Overall, regulated pathways reveal that, under hypoxic conditions, energy production is maximized through nitrate metabolism coupled to other metabolic pathways, enhancing the metabolic state of the cell [52].

Under anaerobic conditions without nitrate supplementation, there was no difference between the wild type and *narQ* or *narP* mutants. However, with nitrate supplementation under anaerobic conditions, 113 differentially expressed genes were detected. Genes upregulated by NarQP in response to nitrate include the glycerol uptake protein GlpF, carbohydrate metabolism proteins (DhaK, Man_2, and Rpe), enzymes involved in nitrate metabolism (NapF, NapH, and NrfA), and hydrogenases (HypD and HyaA). Downregulated genes included the outer membrane protein OmpW, formate dehydrogenase accessory protein FdhE, molybdopterin subunit MoaD, the biofilm extracellular matrix poly-*b*-1,6-*N*-acetyl-D-glucosamine synthesis protein PgaA, and the formate transporter FocA_2. Downregulation of the *pgaABCD* operon, involved in biofilm synthesis, suggests that nitrate may suppress the sessile status and trigger planktonic growth [52].

Deletion of the nap operon (*napFDAGHBC*), which encodes the periplasmic nitrate reductase, caused growth defects under anaerobic conditions, which were greatly amplified with nitrate supplementation. Furthermore, nitrate reduction was reduced in *napFDAGHBC*, *narQ*, and *narP* single mutants, showing that this nitrate reductase is under direct positive regulation of NarQP in response to nitrate.

In mouse infection models, no difference in final lethality was observed between *narQ*, *narP*, and *nap* mutants and the wild type, but death occurred more slowly in mice infected with mutants (12–48 h postinfection), whereas the wild type caused acute death within 12–16 h postinfection. Bacterial survival in mice was also reduced when an inhibitor of nitrate reductase was administered, and the nitrate reductase enzymatic activity itself was diminished. Although NarQP does not appear to regulate any virulence factors directly, the authors suggested that the increased lethality after nitrate treatment results from the population expansion promoted by nitrate [52].

In contrast, NarP was also shown to bind directly to the promoter region of several genes, including dimethyl sulfoxide reductase subunit A (*dmsA*), biofilm synthesis protein (*pgaA*), fine tangled pili major subunit (*ftpA*), carbon starvation protein (*cstA*), and bifunctional metallophosphatase/5′-nucleotidase (*ushA*). Binding to 131 additional genes was detected, but these were not further analyzed. Nitrate stimulated the growth of *A. pleuropneumoniae*, while the five genes listed above are repressed by NarP upon nitrate sensing [54].

It is worth noting that host hormones can also influence *narP* expression indirectly, which is described in a later section [55].

#### 3.2.4. PhoRB

During the acute phase of natural infection in pigs, a transcriptomic analysis showed that *phoR* was upregulated. Even though it is unlikely that porcine lungs are devoid of phosphate, this system might still be important for adaptation to changing conditions within the host [27].

#### 3.2.5. QseCB

Deletion of the *qseBC* operon in *A. pleuropneumoniae*, encoding the TCS QseC-QseB, led to the upregulation of 27 genes and downregulation of 17 genes. Among the downregulated genes were *tonB1* and *hugZ* (both involved in iron acquisition and virulence), *pilM* and *galE* (possibly involved in biofilm formation), and *ccmA*1 (an ABC multidrug transporter) [56].

The system appears to constitute a basal transcriptional regulon, since no specific cognate stimulus has been identified for QseCB [56]. Nevertheless, treatment of *A. pleuropneumoniae* with the mammalian hormones EPI and NE revealed transcriptional responses involving TCS genes. EPI upregulated *qseB* 1.59-fold and downregulated *narP* -2.29-fold, whereas NE downregulates *qseB* -1.51-fold and upregulates *narP* 1.90-fold [55]. Therefore, the QseCB system appears to be positively regulated by EPI and negatively regulated by NE. In contrast, NarQP was differentially regulated: EPI induces *narQ* but inhibited *narP*, while NE induced *narP* and inhibited *narQ*.

Transcriptional analysis also showed that QseCB significantly downregulates the *apf* gene cluster, and biofilm formation was impaired in both *qseBC* and *apfABCD* single mutants. Moreover, *qseBC*, *apfA*, *apfB*, *apfC*, and *apfD* single mutants showed defective anti-phagocytosis of RAW264.7 macrophage, as well as reduced adhesion to and invasion of newborn pig tracheal (NPTr) cells. In mouse infection assays, survival at 120 h postinfection varied across groups, but at 6 h postinfection, bacterial loads in lungs and blood were significantly lower in all mutants compared to the wild type. These findings suggest that the QseCB system and *apf* gene cluster contribute to virulence during the early stages of infection [57].

Finally, the *apf* gene cluster and *qseBC* mutants are more sensitive to acidic and osmotic stress and showed reduced biofilm formation, indicating that QseCB influences stress resistance and biofilm formation by regulating the *apf* gene cluster. Taken together, these results suggest that QseCB controls the expression of the *apf* gene cluster, thereby affecting adhesion, invasion, and the establishment of infection [57].

### 3.3. Glaesserella parasuis

#### 3.3.1. ArcBA

A mutant for the histidine kinase *arcA* showed significant growth defects under anaerobic, but not under aerobic conditions, which is consistent with its homology to two-component systems that respond to redox states. Infected animals challenged with the *arcA* mutant displayed lower clinical symptoms, survived after day 4 postinfection, and recovered from infection symptoms by day 6, whereas complementation and wildtype EP3 strains showed similar survival outcomes. These findings indicate that the ArcBA system is involved in bacterial virulence. Furthermore, loss of *arcA* resulted in higher sensitivity to serum killing compared with the wildtype EP3 strain [29].

Biofilm formation was also compromised in the mutant strain, although the exact mechanism or chemical composition was not assessed. Biofilm formation in microorganisms is a mechanism that allows them to become persistent colonies, enhances their resistance to host immune clearance and antibiotics, and facilitates genetic material exchange [29].

#### 3.3.2. CpxAR

This system, particularly the response regulator CpxR, plays an essential role in counteracting and surviving oxidative, osmotic, and alkaline pH stresses. Mutants with the deletion of either CpxR or the entire CpxAR system showed reduced growth in the presence of macrolide antibiotics such as erythromycin, tilmicosin, and tylosin tartrate. Resistance to erythromycin may be regulated by this system through influencing the expression of the efflux pump genes HAPS_RS09425, which encodes a multidrug-efflux transporter, and HAPS_RS00160 (*acrA*), a member of the resistance-nodulation-cell division (RND) pump family. Accordingly, CpxR contributes to resistance against several macrolides and the aminoglycoside gentamicin [30].

#### 3.3.3. QseCB

Using an old annotation possibly referencing the genomic organization of the system, the system was described as CheY/QseC, whereas the current annotation has it as QseCB (QseC-QseB). A *cheY* mutant, now considered a QseB homolog (Table 3), showed decreased growth, biofilm formation, and survival rate in serum compared to the clinical isolate wildtype EP3 strain. However, the survival rates were similar, with 68.75% in the *qseB* mutant and 71.51% in the wildtype. Agglutination, considered a suspected indicator of virulence, was slower in the mutant compared to the wildtype strain [31].

Both proteins of the QseCB system are involved in biofilm formation, a mechanism associated with virulence [31,32]. Moreover, QseB appears to regulate capsular polysaccharides (CPS) biosynthesis. In a *qseB* single mutant, 11 of the 12 genes on the CPS cluster are downregulated except for *lsgB*. Among these, four encode glycosyltransferases (*lsgB*, *wcwK*, *wcfQ*, and *wbgY*), and one is a precursor of sugar biosynthesis (*capD*); notably, *capD* was the most significantly downregulated. A *capD* single mutant lost the ability to form biofilms but displayed increased adhesion to NPTr cells [33].

QseB was shown to bind directly to the *capD* promoter region. Interestingly, *capD* expression differs between parental and *qseB* mutant strains, both in the presence and absence of EPI. A model has been proposed in which phosphorylated QseB negatively regulates CPS promoters upon EPI sensing [33]. However, in the absence of EPI, *capD* downregulation in the *qseB* mutant was incomplete, and the complemented *qseB* shows an expression pattern similar for *capD* to that of the *qseB* mutant. This aspect should be further investigated to determine precisely how QseB contributes to *capD* regulation, and whether additional two-component systems or elements are involved in the regulatory cascade.

Exposure of the *qseC* mutant to exogenous EPI weakened its ability to sense autoinducer-3 (AI-3) which plays an important role in growth. The specific mechanism has not been described thoroughly yet, but it would shed light on interkingdom communication and bacterial pathogenesis [32]. In addition, QseC contributes to stress tolerance, as the *qseC* mutant showed decrease response to osmotic stress, oxidative stress, and heat shock [32].

The QseCB system may influence iron utilization by regulating the expression of iron uptake genes [32]. Functionally, *qseCB* or *qseB* mutants showed decrease adhesion to porcine alveolar macrophage cells (PAM) and reduced ability to invade these cells. In mouse lung epithelial cells (MLE-12), both adhesion and invasion were also reduced [34]. As expected, the *qseCB* mutant showed a greater decrease in its ability to adhere and invade cells compared to both the parent strain and the *qseC* mutant, suggesting that QseB may still be phosphorylated by a non-cognate histidine kinase or through a metabolic state intermediary [58,59]. As z-stack confocal imaging analysis was not performed, adhesion defects were evident, whereas further imaging would be required to confirm invasion.

Mice challenged with the parental strain displayed severe congestion of alveolar wall capillaries and 87.5% of the animals died. In contrast, both *qseCB* and *qseC* mutants showed only 50% mortality, with reduced lower secondary inflammation in the lungs, and lessened inflammatory damage to the target tissues. Bacterial-induced apoptosis was also greatly reduced in both mutants compared to the parental strain, although the connection this system has to an apoptosis-mediated pathway is uncertain [34].

### 3.4. Mannheimia haemolytica

#### NarQP

Differential expression of two proteins, leukotoxin (Lkt) and FbpA, was seen in a *narP* mutant in response to NaNO_3_ in media. NarP appears to repress Lkt expression directly or indirectly, regardless of NaNO_3_ supplementation. Lkt is a major virulence factor, known to attack bovine macrophages during infections [35]. Unpublished data also supports a link between leukotoxin and NarP regulation, indicating that NarP may select transcriptional regulators from Lkt [36].

FbpA, a periplasmic protein involved in iron acquisition, receives iron from the outer membrane transferrin-binding proteins TbpA and TbpB and transports it to the inner membrane ferric transporters FbpB/FbpC. The authors suggested that regulation of *fbpABC* by NarP may provide a mechanism to sense the bacterium’s location in the host alveoli and facilitate survival in an iron-limited environment [35].

Another periplasmic iron-binding protein of an ATP-transport system, YfeA, is also overproduced in response to NaNO_3_, along with FbpA. This indicates that both are under direct control of the NarQP system. Although these proteins do not participate directly in nitrate respiration, they contribute to the establishment of the system in oxygen-deprived lungs during BPP pathogenesis [37].

Superoxide dismutase (SodA) is also upregulated in response to NaNO_3_. SodA is an important virulence factor in *M. haemolytica*, protecting the bacterium from oxidative damage caused by neutrophils under leukotoxin attack. Its regulation appears to occur indirectly through the NarQP system [37].

Expression of *narP* is upregulated in both pharyngeal swabs and lung washes, with markedly higher expression in the latter. This reflects the pathogen’s need to adapt to low oxygen availability in the lower respiratory tract compared to the pharynx. These findings underscore the complexity of in vivo iron acquisition and suggest that the primary iron scavenger varies depending on both the stage and location of the infection [38].

### 3.5. Pausterella multocida

#### 3.5.1. ArcBA

As expected, this system contributes to growth under anaerobic conditions, which may be important for the pathogenesis of this organism since it causes a spectrum of diseases in a wide range of hosts and must overcome hypoxic environments during infection. Both single mutants, *arcA* and *arcB*, showed reduced biofilm formation as well as decreased resistance to bacterial phagocytosis by murine macrophages. In contrast, deletion of *arcA* significantly reduced recovery in 20% swine serum compared to the wild type, whereas the *arcB* deletion showed no difference from the wild type under the same conditions [42].

Bacterial adhesion to and invasion of swine trachea epithelial cells were also reduced in the *arcA* deletion, and virulence was attenuated when assessed in *G. mellonela* larvae. In mouse models, the wildtype strain killed all mice within 12 h post-challenge, while those challenged with the *arcA* mutant exhibited a survival rate of 60% at the same time point. Bacterial recovery was significantly higher from lungs, brains, and blood vessels of mice challenged with the wild type compared to those challenged with the mutant.

Transcription analysis of the *arcA* mutant revealed 160 differentially expressed genes: 90 upregulated and 70 downregulated. KEGG enrichment analysis of the downregulated genes highlighted pathways related to biofilm formation, tuberculosis, *Salmonella* infection, O-antigen nucleotide sugar biosynthesis, fructose and mannose metabolism, and RNA degradation [42]. Interestingly, two two-component system pathways were also enriched, though further research is required to identify which other systems participate in the same regulatory cascade, whether these are response regulators or histidine kinases, and how these interactions occur.

Deletion of universal stress protein (*uspE*), upregulated by *arcA*, decreased cell adhesion and biofilm formation but increased serum sensitivity and bacterial loads in post-challenge murine models compared to the wildtype strain. Nevertheless, *uspE* did not appear to be a critical virulence factor, since its deletion did not affect lethality at low doses in mice—likely reflecting the intrinsic high virulence of the wildtype strain [42].

#### 3.5.2. QseCB

Phosphate and polymyxin B assays demonstrated that *P. multocida phoP2* is the functional *phoP* gene in this species, based on a complementation of a *Salmonella enterica* serovar Typhimurium *phoP* mutant with either *phoP1* or *phoP2* [39]. However, both genes are annotated with different homologies in NCBI: *phoP2* is annotated as *qseB*, and *phoP1* as *phoB*. Xiao et al. [39] also acknowledge the possibility that *phoP1* corresponds to *phoB*. Therefore, in this review, *phoP2* will be referred to as *qseB* homolog, following its current classification (Table 3).

The *qseB* single mutant exhibited no differences in growth, outer membrane protein (OMP), or lipopolysaccharides (LPS) profiles but did show decreased resistance to polymyxin B in a *P. multocida* background. In a duck infection model, the *qseB* mutant showed a 154-fold higher LD_50_ compared to the parental strain, indicating reduced virulence. Transcriptomic analysis revealed differential expression of 334 genes between the *qseB* mutant and the parental strain, with 161 upregulated and 173 downregulated. KEGG enrichment analysis further showed that these genes were significantly associated with five pathways: bacterial secretion, RNA degradation, protein export, nitrogen metabolism, and ribosome [39].

Ducks immunized with the *qseB* mutant strain showed a survival rate of 54.5% after challenge with the wildtype strain, whereas all control ducks were dead within one week. Based on these results, the authors proposed that the *qseB* mutant could serve as a putative vaccine candidate [39].

QseC has been shown to be under positive selection and may contribute to bacterial adaptation to various niches [40] and to negatively regulate oxidative stress and osmotic pressure resistance [41]. Infection experiments demonstrated that the survival rate of mice exposed to a *qseC* single mutant was significantly higher compared to those infected with the parental or complemented strains; only a weak inflammatory response was detected, and bacterial recovery from the lungs was markedly reduced [41]. Using a *qseC* mutant live strain induced higher levels of cross-reactive antibodies than the inactivated mutant or parental strains. Interestingly, both live and inactivated *qseC* mutants conferred strong cross-protection to mice against multiple *P. multocida* strains, though the live strains showed better results. Thus, the authors propose that it has the potential to be developed as an attenuated vaccine for homologous and heterologous serotypes [41].

Transcriptomic analysis revealed 1245 differentially expressed genes in the *qseC* mutant, with KEGG pathway enrichment analysis highlighting genes related to the outer membrane, ABC transporters, biofilm formation, lipopolysaccharide biosynthesis, and amino acid biosynthesis. Additionally, it was also demonstrated that *qseB* was promoted when *qseC* was deleted, which suggests that another element activates QseB expression [41].

Considering that differential expression reports for *qseC* and *qseB* mutants exist, cross-referencing subsets of differentially regulated genes is possible [39,41]. Unfortunately, reports are in different formats, making it unfeasible to address this thoroughly without reprocessing all datasets for direct comparisons. Nonetheless, a couple of regulation targets could be retrieved from both profiles—downregulated in both were *kdsA* and *tonB*, while *pal* was upregulated. Other genes show opposite regulatory patterns, such as *hyaD*, which was upregulated in a *qseB* mutant but downregulated in a *qseC* mutant, while *lptA* was downregulated in the *qseB* mutant but upregulated in the *qseC* mutant.

This highlights the importance of revisiting early studies that relied on bioinformatics-based functional classification and transcriptome sequencing to ensure proper annotation and cross-study consistency.

The depth of information varies across species, reflecting an uneven research coverage rather than biological absence; while *P. multocida* and *A. pleuropneumoniae* have been analyzed extensively, fewer experimental studies are available for *H. influenzae*, *M. haemolytica*, and *G. parasuis*. Accordingly, we summarize all existing data and highlight cross-species patterns where direct comparisons were possible (Table 2).

**Table 2:** Major two-component systems characterized in *Pasteurellaceae*. Details of the listed systems include their known environmental inputs, validated regulatory targets, and experimentally demonstrated contributions to virulence. Two-component systems are listed as histidine kinase–response regulator (HK–RR) pairs. All shown HKs are membrane-associated sensors with predicted transmembrane domains, reflecting their roles in detecting extracellular or periplasmic cues. Systems are ordered by number of Pasteurellaceae species for which experimental or transcriptomic evidence is available, highlighting those most functionally characterized. Species are shown only where functional or transcriptomic data exist. Conservation patterns across *Pasteurellaceae* are provided separately in Figure 2.

TCS	Sensor Type/Signal	Core Regulon/Key Targets	Virulence-Related Functions	Species with Experimental Evidence	References
ArcBA	Redox state, anaerobiosis	Formate dehydrogenase, fumarate reductase, LOS glycosyltransferases, respiratory chain genes (*fdxH*, *fdxI*, *fdhE*, *ndh*, *lldD*), metabolic pathways	Serum resistance, oxidative stress survival, biofilm formation, adhesion/invasion, persistence in vivo	*H. influenzae*, *A. pleuropneumoniae*, *G. parasuis*, *P. multocida*	[16,17,18,22,23,29,42]
QseCB	Signal unclear (no clear AI-3/EPI/NE sensing except species-specific effects), cold shock, ferrous iron	Adhesins, LOS biosynthesis, *apf* pilus cluster, CPS cluster, iron uptake	Biofilm formation, adhesion/invasion, modulation of stress responses, attenuated virulence, vaccine potential	*H. influenzae*, *A. pleuropneumoniae*, *G. parasuis*, *P. multocida*	[19,20,31,32,33,34,39,40,41]
NarQP	Nitrate/nitrite, anaerobic respiration	*napFDAGHBC* operon, nitrate metabolic enzymes, biofilm genes (*pgaA*), electron transport chain	Growth advantage in hypoxia, modulation of biofilm vs. planktonic state, metabolic rewiring, slower lethality when mutated	*H. influenzae*, *A. pleuropneumoniae*, *M. haemolytica*	[21,35,36,52,53,54,55]
CpxAR	Membrane envelope stress, temperature stress	Type IV pili genes, capsule export genes (*cps*), *wecA*, efflux pumps	Capsule production, heat-stress response, adhesion, serum resistance, macrolide resistance, virulence in mice	*A. pleuropneumoniae*, *G. parasuis*	[24,25,26,27,28,30,50,51]
PhoRB	Phosphate limitation	phoR-driven responses; likely phosphate uptake genes	Colonization during infection despite phosphate availability; role still emerging	*A. pleuropneumoniae*	[27]

## 4. Challenges in TCS Characterization

Overall, it is difficult to infer the exact functions of two-component systems even within the same family, notwithstanding their high sequence homology. A major challenge is that signals sensed by histidine kinases are often unknown; while homology can sometimes provide clues, this is not always reliable. Moreover, members of the same TCS family may respond to more than one stimulus and have very different regulatory cascades across closely related species [16,17,18,19,20,22,23,29,31,32,33,34,39,41,42,49,55,56,57].

**Figure 3:**
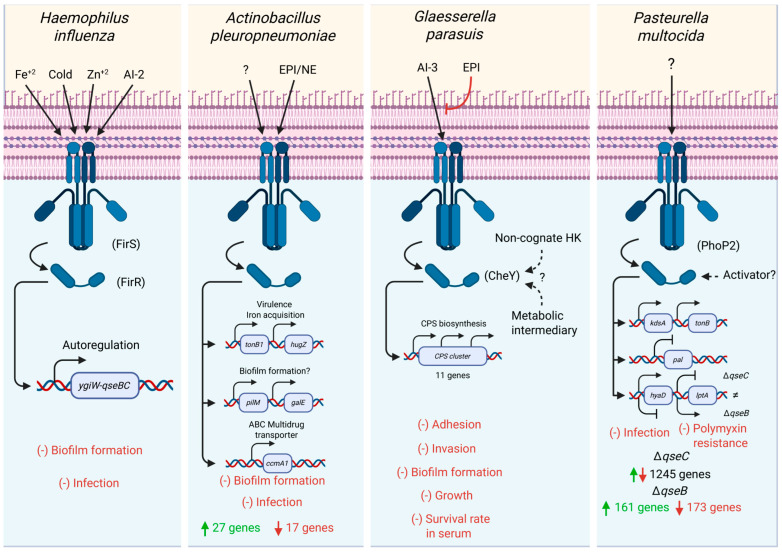
Comparative regulation of the QseCB two-component system across four *Pasteurellaceae* species. Each panel shows the cognate histidine kinase (QseC) and response regulator (QseB), the major input signals reported, and the principal regulatory targets identified in each species. Arrows indicate activation of transcriptional targets or autoregulation; dashed lines indicate predicted or non-cognate interactions. The number of differentially expressed genes in Δ*qseC* or Δ*qseB* mutants is shown, where available. Negative phenotypic effects are indicated in red. HKs’ historical annotation variants are noted in brackets (Table 3).

An important example is the concept of the regulog, a conserved core of regulated elements [60]. The best defined regulog so far in *Pasteurellales* is for the NarP response regulator, with conserved elements identified in *H. influenzae*, *P. multocida*, *A. pleuropneumoniae*, and *G. parasuis* [61]. In contrast, no other TCS protein has such a defined regulog, even though several transcriptional analyses have been performed. This highlights the need for more comprehensive cross-species comparison of transcriptional outputs. Among *Pasteurellaceae*, ArcBA and QseCB are best characterized at the transcriptional level (Figure 3) [16,17,18,19,20,22,23,29,31,32,33,34,41,42,49,55,56,57].

This also underscores the need for a consensus on how TCSs are named and classified—by function or homology. For example, QseCB has been referred to as FirSR in *H. influenzae*, and *qseB* has been named *phoP2* in *P. multocida* [19]. Such inconsistencies hinder the ability to collect information systematically and to identify conserved regulogs across species.

**Table 3:** Standardized nomenclature for two-component system proteins with conflicting annotations. Current annotations used in the manuscript are shown alongside historical or alternative gene/protein names reported in the literature, with corresponding species and references. This table resolves inconsistencies in naming across studies and highlights the preferred terminology adopted here.

Current Annotation	Alternative Name in Literature	Species	Reference
PhoB	PhoP1	*Pasteurella (P.) multocida*	[39]
QseC	FirS	*Haemophilus (H.) influenzae*	[19]
QseC	PhoP2	*Pasteurella (P.) multocida*	[39]
QseB	FirR	*Haemophilus (H.) influenza*	[19]
QseB	CheY	*Glaesserella (G.) parasuis*	[31]

Another striking observation is that, even when the same stimulus is recognized (e.g., ArcBA sensing redox states), the downstream responses and related virulence factors vary widely among species [16,17,18,22,23,29,42]. This divergence underscores the rewiring of regulatory cascades within the family and raises some key questions: What are the drivers of this regulatory cascade rewiring in closely related pathogens? Although direct crosstalk between TCSs has not been systematically evaluated in vivo, could there be non-cognate interactions that trigger regulatory cascades in specific environmental conditions?

Finally, although the search for TCS inhibitors as antimicrobials is not as active as it was a decade ago, these systems remain attractive targets, since they are absent in higher eukaryotes [1,2,62]. Coupling TCS inhibitors with existing therapies could improve efficacy by disarming pathogens, preventing them from sensing and adapting to host environments.

Additionally, among the species reviewed, *P. multocida* encodes the highest number of TCSs, which may reflect its broad host range and exposure to more variable environments [42]. In contrast, obligate or niche-restricted pathogens often retain fewer TCSs, as stable host-associated lifestyles reduce the need for extensive environmental sensing [2,62,63]. Compared with other *γ*-proteobacteria, *Pasteurellaceae* as a whole carry reduced TCS repertoires, consistent with their opportunistic but host-adapted lifestyles [62]. These patterns suggest that TCS content is shaped, at least in part, by ecological niche breadth and the selective pressures associated with host adaptation. Experimental and phylogenetic work will be required to determine whether these differences represent divergent evolution within lineages, convergent adaptation to similar pressures, or horizontal gene transfer.

## 5. Emerging Computational Approaches

**Figure 4:**
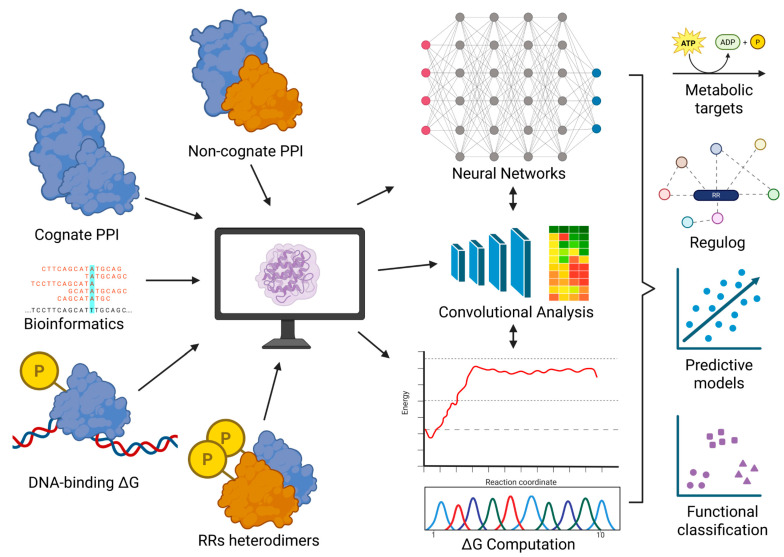
Emerging computational strategies for understanding two-component systems. Structural and energetic features of cognate and non-cognate protein–protein interactions, DNA-binding affinity (ΔG), and potential response regulator heterodimers can be modeled through molecular dynamics and docking. Bioinformatic motif discovery and regulog comparison provide genomic context. These diverse data streams can be integrated through convolutional or neural network-based analyses to predict regulatory motifs, metabolic targets, and system-level classifications across species.

The advent of high-performance computation, artificial intelligence, and machine learning has introduced powerful tools to dissect TCS specificity and regulatory cascades. Molecular dynamics and binding free energy calculations can provide quantitative predictions of HK-RR interactions and RR-DNA binding [64], potentially revealing non-cognate partnerships and crosstalk [65,66]. Machine learning approaches could recognize subtle patterns across transcriptomes that define regulogs, even when not obvious from homology alone [60]. Another possible direction using this same approach is to check whether there are active response regulator heterodimers that can bind to different sequences and respond to specific environmental conditions, as in vivo infection might be (Figure 4).

Structural approaches also hold promise. Clusterization by structural homology, coupled with sequence alignments, could also improve functional family classification. Convolutional neural networks—already used in pipelines such as AlphaFold to recognize spatial patterns—could be applied to HK and RR structures to distinguish proteins with similar sequences but divergent three-dimensional configurations [67,68]. Such integration of bioinformatics, computational biochemistry, and cheminformatics could eventually enable reconstruction of regulatory cascades and prediction of metabolic targets with unprecedented accuracy.

Tools such as AlphaFold2, AlphaFold-Multimer, and RoseTTAFold can model HK and RR structures with near-experimental accuracy, enabling prediction of interaction interfaces, domain complementarity or compatibility, and the effects of point mutations [67,68,69]. For complexes, predictive accuracy decreases; HK-RR complexes and RR-DNA complexes have not been thoroughly sampled, and predicted structures may fail to reflect dynamic conformational changes or activation states (e.g., phosphorylation). Computational approaches such as quantum mechanics/molecular mechanics (QM/MM) can model the energetics of phosphorylation with high precision, but they remain computationally expensive and technically specialized [70,71].

Graph-based machine learning, convolutional neural networks, and structure-based clustering of AlphaFold models can group proteins with similar folds despite low sequence identity; large-scale studies have used AlphaFold predictions to cluster millions of structures and uncover new families and folds, demonstrating the utility of this approach for identifying remotely related regulatory proteins [72,73]. The graph neural network-based sequence-only interaction predictor xCAPT5, which combines large protein language model embeddings with convolutional Siamese networks, can infer protein–protein interactions [74]. However, these models require large, high-quality training sets, and HK-RR interactions involve features (transmembranal, cytosolic, periplasmic, or external domains, as well as dimerization states) that may be underrepresented in generic protein–protein datasets, increasing the risk of overfitting to known families.

Molecular dynamics simulations can quantify binding free energies between RR and promoter DNA to predict transcriptional outcomes, and network-analysis approaches integrating ChIP-seq or RNA-seq can reconstruct TCS-driven regulons. Structure prediction remains less reliable for highly flexible sensory domains, molecular dynamics simulations are computational demanding, and machine learning pipelines depend heavily on data quality and may fail when experimental datasets are scarce.

Despite these caveats, machine learning, artificial intelligence, and computational biophysics offer scalable methods to generate testable hypotheses and prioritize experimental targets for validation. To close the loop between prediction and function, computationally inferred HK-RR pairs or RR-DNA interfaces could be tested experimentally. HK-RR phosphotransfer can be validated using in vitro phosphotransfer assays, in vivo fluorescence resonance energy transfer (FRET), or autophosphorylation kinetics with purified proteins [8,11,75,76,77]. RR-DNA binding predictions can be examined via EMSA, DNAse footprinting, or promoter–reporter fusion in relevant growth conditions [54,78,79,80]. RNA-seq or ChIP-seq-derived network predictions can be verified through gene deletions or complementation and monitoring transcriptional or virulence phenotypes [81,82,83]. Validated interactions can then be fed back into training datasets or structural models to improve prediction accuracy and reduce false positives. Such combined pipelines—computational prioritization followed by targeted biochemical and genetic testing—provide a feasible route to experimentally resolve TCS interaction specificity and downstream regulatory cascades in cases where large-scale biological assays would be otherwise impractical.

## 6. Conclusions

Two-component systems are central regulators of virulence and adaptation in *Pasteurellaceae*, yet their precise roles remain difficult to define. Despite high sequence homology among histidine kinases and response regulators, the regulatory outputs often diverge widely between species. This divergence is partly due to the frequent lack of knowledge about the signal sensed by the histidine kinase, the possibility of multiple stimuli per system, and the absence of clearly defined regulogs beyond NarP. Among the systems reviewed, ArcAB and QseCB are the best characterized transcriptionally, but even here, no conserved regulon has been resolved across species.

Another major challenge is inconsistency in TCS annotation, where identical systems are named differently in various databases or publications (e.g., QseC referred to as FirSR in *H. influenzae* or *qseB* referred to as *phoP2* in *P. multocida*). This complicates cross-study comparisons, and integrations of structural as well as sequence-based classifications are necessary.

Attempting to mutate every regulatory element within a species—or across multiple species—is ultimately a Sisyphean task: it is resource-intensive, limited in scope, and unlikely to capture the divergent responses observed among *Pasteurellaceae*, let alone across bacteria. Computational modeling of regulatory cascades and protein–protein interactions offers a scalable way to generate hypotheses and prioritize experiments, in a predictive framework.

Beyond functional characterization, a comprehensive evolutionary analysis of gene organization could clarify why some TCSs are widely conserved while others occur sporadically or as duplications. Comparative phylogenetic and synteny analyses across *Pasteurellaceae* genomes may reveal whether these patterns reflect divergent or convergent evolution, adaptation to specific hosts or niches, or horizontal gene transfer. Such approaches would help explain how TCS signaling has been shaped over time and why closely related species display distinct regulatory responses.

Advances in computation, including AI-driven structural prediction, molecular dynamics, and machine learning applied to transcriptomic data, promise to accelerate the discovery of conserved regulogs and regulatory motifs. These approaches may soon allow us to move beyond single-system characterization toward a systems-level understanding of TCS regulatory networks across *Pasteurellaceae*.

## Data Availability

No new data were created or analyzed in this study. Data sharing is not applicable to this article.

## Abbreviations

The following abbreviations are used in this manuscript:

TCS	Two-component system
HK	Histidine kinase
RR	Response regulator
CPS	Capsular polysaccharides
EPI	Epinephrine
NE	Norepinephrine
RND	Resistance-nodulation-cell division
NPTr	Newborn pig tracheal cells
AI-3	Autoinducer-3
PAM	Porcine alveolar macrophage
MLE-12	Mouse lung epithelial cells
Hib 10211	*H. influenzae* type b ATCC 10211
NTHI	Non-typeable *H. influenzae*
NT127	*H. influenzae* clinical isolate
LOS	Lipooligosaccharide
ROI	Reactive oxygen intermediates
AI-2	Autoinducer-2
OMP	Outer membrane protein
LPS	Lipopolysaccharides
Lkt	leukotoxin
PPI	Protein–protein interaction
QM/MM	Quantum mechanics/molecular mechanics
